# The prince and the scientists: The ecology of scientific leadership

**DOI:** 10.1038/s44319-026-00964-5

**Published:** 2026-10-08

**Authors:** Vincent Laudet

**Affiliations:** 1 Okinawa Institute of Science and Technology, Onna, Japan; 2 Academia Sinica, Taipei, Taiwan; 3 IRL CNRS-OIST EARLY, Onna, Japan

**Keywords:** Careers, History & Philosophy of Science

## Abstract

Scientific institutions pose a peculiar leadership challenge: their ultimate purpose, scientific discovery, cannot be commanded or predicted. I argue that scientific leadership is less about exercising authority than about cultivating the trust, diversity, interactions and intellectual freedom from which scientific excellence can emerge.

**Figure:**
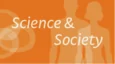

Every time I participate in the evaluation of a laboratory, an institute, or a university, the scenario is essentially the same. The leadership team presents their strategy, priorities, challenges, and ambitions, followed by discussions about recruitment, funding, governance, and long-term vision. All of this is essential for a critical and fair assessment.

But, over the years, I have developed another habit. I always try to find a moment to talk with the administrative staff, technicians, or students. No formalities. No presentation. Just a conversation over coffee in a hallway or in a lab. And, very often, that is when I begin to understand what is really going on: whether people trust their leadership, take pride in their institution, collaborate naturally, are encouraged to take risks, and feel part of a scientific community rather than simply an organization.

These conversations do not necessarily contradict what the leaders say. They simply tell another part of the story, one that is harder to measure or formalize, yet equally essential to understanding why some institutions generate exceptional science, while others, despite comparable resources, remain merely good. Perhaps because I have not always experienced great leadership over my career, one question has puzzled me for years: why do institutions operating under similar rules, with comparable resources and equally talented scientists, achieve such radically different levels of scientific excellence?

Leadership has been studied extensively, including in academic and scientific settings. Yet, the peculiar challenge of leading a scientific institution deserves special attention. Scientific leaders exercise considerable authority over organizations, the ultimate purpose of which, discovery, is something they cannot command or even predict. This matters a lot because poor leadership can affect creativity, trust, and the scientific trajectory. Understanding what makes a good scientific leader therefore deserves at least as much attention as understanding what makes a good scientist. This is the question I want to explore here.

“Understanding what makes a good scientific leader therefore deserves at least as much attention as understanding what makes a good scientist.”

## A scientific institution is not a business

For a long time, leadership was associated with the idea of the “great man”; a providential figure whose personality, authority, and charisma were enough to guarantee success. This vision has always reminded me of my father. Tall and imposing, he naturally commanded the room and always found himself in a position of strength during negotiations. Needless to say, when I came to him to have my Latin translations corrected, the balance of power was somewhat unequal. There was something almost theatrical about this display of authority, but it reflected a deeply rooted conception of leadership: the leader leads because he is a “great man”, and yes, the gender stereotype is very much part of the concept.

Leadership theories have fortunately changed considerably since, placing much greater emphasis on human relations, motivation, collective vision, and the ability to inspire rather than simply command. But scientific institutions pose a more fundamental problem: can they actually be led in the same way as other organizations? An army seeks to win a battle. A company aims to make profits, develop a product, or conquer new markets. A government pursues a political agenda. The objectives may be ambitious, complex, and sometimes contradictory, but they remain identifiable. A scientific institution pursues a goal of a radically different nature. Its purpose is to generate something that does not yet exist. A true discovery is, by definition, unpredictable. It may seem obvious, but it is perfectly captured by Einstein’s famous quip: “If we knew what it was we were doing, it would not be called research, would it?”

This simple difference profoundly changes the nature of leadership. Scientists are recruited precisely because they think for themselves, challenge accepted ideas, and follow new paths that nobody had anticipated before. We cannot simultaneously ask them to be fiercely original in their science and docile within their institution. This creates another fundamental difference. A general commands, and a CEO imposes a decision. A scientific leader has such formal authority too, but authority alone is a remarkably poor instrument for producing science. Researchers can be instructed to attend a meeting, follow a procedure, or respect a budget; they cannot be instructed to have an original idea. Scientific leadership, therefore, depends much more on example, persuasion, and the ability to articulate a vision that scientists eventually make their own. The role of a scientific leader is not to tell researchers what to think or what to investigate, but to create an environment that allows them to do this by themselves.

“Researchers can be instructed to attend a meeting, follow a procedure or respect a budget; they cannot be instructed to have an original idea.”

I witnessed the flaws of this managerial mindset early in my career. A new secretary general, hired from the private sector as a master organizer, applied corporate methods to the institute. He optimized procedures, streamlined decisions, and boosted efficiency. It was a total disaster. He brilliantly managed the organization while missing its core purpose: generating knowledge. A scientific institution cannot be optimized like a production line. This experience made me realize that uncertainty, intellectual freedom, diversity of approaches, and risk-taking are not flaws that scientific leaders should try to eliminate. They are part of the process by which discovery occurs.

Niccolò Machiavelli understood that even the most powerful Prince could never entirely master fortuna. His task was not to abolish uncertainty, but to prepare for it. I use Machiavelli here deliberately as a counterpoint rather than as a model. His Prince is concerned primarily with acquiring, exercising, and preserving power; the scientific leader faces almost the opposite challenge: how to use power to foster the independence and creativity on which science depends. Ironically, despite these fundamental differences in purpose, scientific leaders still too often adopt the codes of the Prince: hierarchy, displays of authority, and the enduring temptation of the “great man” model of leadership. Such displays may reinforce authority, but they contribute remarkably little to science.

## Scientists do not follow titles; they follow peers

The head of a scientific institution is not expected to be the best or most productive researcher in the institution. Their role is not to do science in place of others, nor to guide the scientific choices of dozens or hundreds of teams. And yet, I am convinced that a great scientific leader must be an excellent scientist.

At first sight, this may seem paradoxical. A football coach no longer needs to score goals, but having practiced the same craft provides an intimate understanding of the people they lead and of the difficulties they face. This is also where scientific leadership departs from Machiavelli’s Prince. Political authority can be inherited, acquired, or conferred by office. Scientific legitimacy cannot. No appointment as President, Provost, or Director can suddenly create it. It has to be earned through the practice of science.

Researchers do not expect their leaders to tell them what experiments to perform or which questions to pursue. But they do expect them to understand what science actually means. A credible scientific leader has experienced failed experiments, rejected papers or grants, years of work leading nowhere, but also the incomparable thrill of discovery. This shared experience forms the basis of what I would call epistemic legitimacy. It is not an administrative authority. It is the recognition by a community that its leader understands the nature of science from the inside and what is at stake when decisions affecting it are made.

This legitimacy is necessary, but obviously not sufficient. The history of universities and research institutions is full of exceptional scientists who became mediocre Presidents or Provosts. Being able to advance science and being able to lead a scientific community are two profoundly different talents. Scientific excellence provides legitimacy; it does not magically provide leadership.

But there are critical moments when genuine scientific judgment becomes indispensable. Should an institution continue supporting a laboratory that publishes little but is extraordinarily creative? Should it recruit a little-known young scientist with great potential rather than a safer, more consensual candidate? Such decisions cannot be reduced to performance indicators, publication counts or management dashboards. They require genuine scientific judgment. This is perhaps the ultimate responsibility conferred by epistemic legitimacy. A scientific leader must recognize exceptional science long before it becomes obvious to everyone else.

## The institution as an ecosystem

When I look at a major scientific institution, I do not see an organization. I see an ecosystem. An ecosystem is more than the sum of the species that make it up. Its richness, stability, and resilience depend largely on the interactions connecting them, from food webs and symbioses to keystone species and ecological niches.

The same is true of a scientific institution. A president, director, or dean does not simply lead a collection of independent researchers. They foster an ecosystem in which researchers, students, engineers, technicians, administrative staff, technology platform managers, grant writers, fundraisers, and policymakers interact. The quality of an institution depends as much on these interactions as on the individual talents it contains.

This is why recruiting the best researchers is not enough to build the best institution. A coral reef is not remarkable because it brings together the “best species”. It is remarkable because it has built an extraordinarily rich network of interactions. I learned this lesson from Jamshed R. Tata, the British molecular biologist, who spent six months in my laboratory when I was a young group leader. Before leaving, he told me something I have never forgotten: “Your lab is excellent, but you have ten Zidanes in your team. That cannot work. You only need two or three goal scorers. You need more diversity, otherwise your team will have no structure and no soul.” He was right. Excellence is not about assembling as many stars as possible. A great team, like a great ecosystem, depends on diversity, complementarity, and the interactions between its members.

Ecologists refer to “keystone species”: species whose disappearance can profoundly destabilize an ecosystem. Scientific institutions have their own keystone species. They are not necessarily the most famous researchers. It may be a brilliant engineer who keeps an indispensable platform running, or an administrator who somehow always finds a solution when everyone else is stuck. Their importance often bears little relation to their position on the organizational chart.

Other organisms, such as beavers or corals, are called “ecosystem engineers” because they transform their environment and, in doing so, reshape the opportunities available to other species. Scientific leaders can play a similar role. Through decisions about recruitment, resources, infrastructure, organization, and even physical space, they reshape the landscape in which science takes place. Creating a department, opening a new research area, establishing a technology platform, or constructing a building are therefore not merely administrative decisions. They alter the scientific habitat, bringing some people and disciplines into contact, separating others, and creating new possibilities that may persist for decades. Such decisions require judgment about when an institution should expand into new territory and when it should resist the temptation to grow simply because resources are available. Leaders cannot determine which interactions will occur or what discoveries will emerge from them. But they can profoundly influence which interactions and discoveries become possible.

But an ecosystem cannot be shaped from the top alone. The culture created by a President or Director ultimately has to be reproduced at every level of the institution, in departments, administrative units and, most importantly, individual research groups. A President may promote openness and intellectual risk, but this means little to a doctoral student working in a laboratory ruled by fear. Conversely, excellent local leadership can preserve creativity even when the wider institution is struggling. I have also witnessed the opposite process several times: poor leadership at the top gradually contaminating an entire institution, as distrust, caution, and dysfunctional practices spread from one level to the next. The effects of leadership therefore propagate through an institution much like energy through a food web: what happens at one level affects the functioning of many others. The task of those at the top is not only to lead well themselves, but to create incentives and examples that allow good leadership to diffuse throughout the institution.

The ecological analogy also illuminates another critical idea: the importance of margins. Evolutionary novelty often emerges at the periphery, where small, partially isolated populations escape some of the homogenizing forces acting at the center and are free to explore different evolutionary trajectories. Scientific institutions need similar margins. The most effective systems are never fully optimized. They retain unproductive margins of time, funding, and intellectual space to explore an unlikely idea, have a discussion without an immediate objective, launch a risky project, or simply think. To a manager, this may look inefficient. In science, it is crucial as it is often here that novel ideas emerge. Like natural ecosystems, scientific institutions derive resilience from diversity, redundancy, and components that may appear unnecessary until circumstances change.

We can thus appreciate how much a leader’s mission extends beyond the day-to-day administration. Their role is to preserve diversity, protect the key components, maintain flexibility, foster interactions, and create the conditions in which new properties can emerge. For, ultimately, scientific excellence is itself an emergent property. It is not the sum of an institution’s best researchers. It arises from the interactions they develop, the ambition they share, and the environment in which they operate. The leader’s role, therefore, is to cultivate the ecosystem that makes this excellence possible.

“Scientific excellence is itself an emergent property.”

## Trust is the invisible infrastructure of science

If we accept this ecological view of scientific institutions, one question immediately becomes central: what holds this ecosystem together? When a university or research institute seeks to strengthen its excellence, the first instinct is usually to invest in what is visible: new buildings, equipment, or technology platforms. All these investments are useful, often indispensable. But I have gradually come to believe that the most important infrastructure of a scientific institution is invisible. It appears on no organizational chart, is listed in no budget, and is never the subject of an official inauguration. This infrastructure is trusted.

Trust is often regarded as a quality of human relationships. I believe it is a major determinant of scientific performance. Trust accelerates the circulation of ideas, facilitates collaborations, and allows a young doctoral student to knock on a team leader’s door to propose a crazy idea. It allows researchers to share preliminary results or admit that an experiment has failed. These situations may seem trivial, but they are the fabric of everyday research, and this is often where discoveries begin.

When trust disappears, everything becomes more difficult. Researchers become cautious, information circulates less freely, collaborations decline, and procedures multiply. I have often thought that bureaucracy develops like a scar: it appears when trust declines and attempts to replace it with administrative control of procedures that previously functioned through human relationships. The less trust there is, the more rules are introduced; the more rules are introduced, the more trust disappears.

Trust also differs from other forms of infrastructure in one crucial respect. A leader can decide to construct a building, fund a platform, or hire an engineer. But no President can announce that, starting Monday, the community will trust its leadership. Epistemic legitimacy does not guarantee trust, but it provides one of its foundations. Trust is then built slowly through hundreds of apparently insignificant decisions: a promise kept, a difficult decision explained, a mistake acknowledged, a disagreement handled honestly. And it is profoundly asymmetric: it may take years to build and only weeks to destroy.

Machiavelli famously asked whether it was better for a Prince to be loved or feared, concluding that, if one could not be both, it was safer to be feared. That may be an effective principle for securing obedience. It is a disastrous one for producing science. Yet, fear can be as tempting to a weakened leader as flame is to a moth. When trust and legitimacy begin to erode, the temptation is to compensate with authority: more rules, more demonstrations of power. But this only accelerates the process. A leader can compel a researcher to attend a meeting or complete a form. No leader can compel a researcher to have an idea. By trying to restore authority through fear, a weakened leader may simply dig their own grave faster.

This is why scientific leadership is profoundly political, in the noblest sense of the term: it is the art of bringing a human community together around a shared project. Researchers may accept a decision because it is imposed on them. But they will only take risks, share ideas, and give their best if they choose to commit to the collective project that the decision serves. Authority may secure compliance. Trust is what makes a scientific community possible. If a scientific institution is an ecosystem, trust is perhaps its energy: the invisible force that allows its different components to interact and cooperate. Without it, all the components may still be there, but the ecosystem no longer functions as a whole.

“Authority may secure compliance. Trust is what makes a scientific community possible.”

## How should we judge a scientific leader?

If scientific leadership matters as much as I have argued here, then we should probably take it much more seriously as a subject for reflection, teaching, and research. We devote immense effort to understanding how to evaluate researchers, select projects, optimize funding, or measure scientific impact. Yet, we devote surprisingly little attention to how the people entrusted with leading the institutions where science is produced are selected, supported, and evaluated.

There is a strange asymmetry here. Early-career researchers are scrutinized relentlessly. Their projects are evaluated, their papers counted, their performance compared and, as a result, their careers can sometimes be abruptly interrupted. Scientific institutional leaders, by contrast, are often treated with remarkable leniency. This may be particularly true at the level of governing boards, whose members are themselves senior leaders accustomed to the difficulties, compromises, and occasional failures of leadership. It is a small world, and understanding one’s peers can easily turn into excusing them. Curiously, the same generosity is not always extended to younger scientists. Being indulgent with those who hold power and demanding with those who do not is certainly a comfortable position. I am not sure it is one that is intellectually or morally satisfying. Yet, the asymmetry of consequences runs in precisely the opposite direction. A young scientist can make a bad decision and learn from it. A poor leader can damage an entire institution for years.

This does not mean that we need another layer of metrics, rankings, or bureaucratic evaluation. Quite the opposite. Leadership is not easily captured by a dashboard. How does one measure whether a leader has increased trust, encouraged intellectual risk, created unexpected interactions, or made an institution more ambitious? Some of the most important effects of leadership may only become visible years later. But the difficulty of measurement should not become an excuse for the absence of judgment.

Nor should we assume that an excellent scientist will somehow become an excellent leader by osmosis. Scientific leadership is a skill that can be learned, discussed, criticized, and improved. But leadership can also be a surprisingly lonely exercise, particularly when difficult decisions have to be made. Leaders should therefore be encouraged to surround themselves with trusted peers who can challenge their assumptions, offer different perspectives, and help them prepare important decisions before they are made. Future leaders should be mentored before they are appointed, and their performance discussed with the same rigor, honesty, and goodwill that we expect when discussing science itself.

There is, finally, one criterion that may be even more demanding. Machiavelli’s Prince is ultimately concerned with acquiring power, exercising it, and preserving it. A great scientific leader should perhaps aspire to the opposite: to build an institution whose excellence no longer depends on their presence. I directed the Institute of Functional Genomics at the ENS de Lyon for eight years. After I left to take another position, nobody from the institute ever called me to ask how this or that should be handled. Someone once asked me whether I found this upsetting. In fact, I was delighted! To me, it meant that we had built an institute strong enough to function perfectly well without me. Perhaps this is the ultimate achievement of scientific leadership: not to become indispensable, but to leave behind a community that remains ambitious, creative, cohesive, and capable of renewing itself after its leader is gone.

Science has taught us to understand living systems through interactions, complexity, and emergence. Perhaps it is time to apply the same way of thinking to the institutions in which we practice it. Great discoveries depend on exceptional scientists, but exceptional scientists also depend on the communities we build around them.

## Supplementary information

Peer Review File

