# Supplementary material for: The prince and the scientists: The ecology of scientific leadership
Source: EMBO Rep. 2026 Oct 8;27(21):6093–6. doi: 10.1038/s44319-026-00964-5 (PMC13743769; doi:10.1038/s44319-026-00964-5)
Supplement: Supplementary file 1 [file 44319_2026_964_MOESM1_ESM.pdf]

EMBO reports

The prince and the scientists

Vincent Laudet

Corresponding author(s): Vincent Laudet (Vincent.Laudet@oist.jp)

Review Timeline:

Submission Date:	29th Sep 26
Accepted:	1st Oct 26

Editor: Holger Breithaupt

Transaction Report: This article was editorially reviewed.
